# Supplementary material for: “The world is not a safe place”: Representations of emotional distress, coping, and survival among young adults in South Africa
Source: PLOS Ment Health. 2025 Jul 31;2(7):e0000394. doi: 10.1371/journal.pmen.0000394 (PMC12798508; doi:10.1371/journal.pmen.0000394)
Supplement: Attachment — Submitted filename: Coping & Survival Response to Reviewers.docx [file pmen.0000394.s004.docx]

Dear Dr. Montague-Cardoso,

Thank you so much for the opportunity to revise our manuscript titled *“The world is not a safe place”: Representations of Emotional Distress, Coping, and Survival among Young Adults in South Africa*. The reviewer comments have been incredibly helpful; by integrating their feedback, we feel we have produced a stronger contribution to the literature.

Please find our response to each reviewer in turn, in the table below.

We look forward to hearing from you in due course.

With best wishes,

Sorcha Ní Chobhthaigh

University College London, UK

Email: [sorcha.nichobhthaigh.21@ucl.ac.uk](mailto:sorcha.nichobhthaigh.21@ucl.ac.uk)

____________________________________________________________________________________

**Reviewer 1**

Reviewer #1: As outlined by the authors it would not be suitable to make the data for this study openly accessible. This article is well written, and the research is ethically and methodologically rigorous. I have attached my detailed feedback on the article in the attached document.

Thank you for the opportunity to review this really interesting and important study. It develops a novel and creative approach to studying youth mental health in resource-limited settings in South Africa. The comments that follow are aimed at supporting the development of this work. At present all of the ‘pieces’ are in place, however they do not always fit together, or at least would benefit from more reflective discussion and analysis from the authors.

Thank you so much for taking the time to review our paper and for providing such thoughtful feedback. We greatly appreciate the encouragement to explicitly acknowledge reflections and conversations which had shaped this piece, but we had not included previously when prioritising space.

1. There is an inherent tension in this paper – it argues for the need to decentre ‘limiting diagnostic categories’ and ‘Western-bound mental health interventions’ and concepts, however it uses these concepts, worldviews, and diagnostic categories in it’s analysis. For instance, the discussion on the contextually-grounded themes are analysed through the language of ‘risk’, PTSD, (individualistic) ‘cognitive thinking patterns’, self-regulation, (the individual-centred) empowerment process model etc.. More broadly, I wasn’t clear (until p. 25) as to why this work was framed through the lens of trauma – for instance, would these youth identify their contexts as ‘traumatic’, what does labelling these contexts as traumatic *do* (for the youth, for the interventions etc.) – in some ways it could be argued that it individualises the socio-political/-historical, and I would imagine some of these youth would not want to identify as traumatised. I appreciate why this translation (between knowledge systems) is likely needed – i.e. the targeted audience for this paper is mental health professionals – but in order to meaningfully recognise these young people’s worldviews, more conceptual linking/bridging is needed from the authors upfront – *why* is it useful (and for who!) to label chronic oppression as trauma, in what ways might this still be limiting/harmful, how are the authors navigating tensions related to knowledge translations between the young people’s grounded experiences and Western mental health (individualistic) concepts and worldviews?
2. Regarding the latter I wonder if there would be greater opportunity for clearer critical analysis if the findings section solely focussed on the young people’s conceptualisations, and the ‘linking’ to mental health terminology was contained to the discussion section.

Thank you so much for this thoughtful recognition of our efforts to hold a dual perspective – we have added explicit recognition of this to the introduction, and we have also added a comment on this inherent tension to the reflections section. To your second point, we have tried to balance the need for centering of young people, with the need to ‘bring others along’ by including some brief mention of connection to mainstream paradigms in the analysis. However, we hope that with our expanded discussion we are able to highlight the necessity of centering young people’s voices.

3. The pervasiveness of ‘the individual’ lies at the heart of this tension in regard to knowledge translation, and more engagement with the literature on agency would support a deeper analysis of this tension. For instance, there is a rich feminist and post-/de-colonial body of literature on conceptualising agency through more relational/collectivist/new materialist framings. Much of this work would critique the identification of agency as ‘choice’, ‘action’, and the individual-centred ‘empowerment process model’ (in the conceptual framework and discussion sections). Even if young people use the language of ‘choice’ and ‘empowerment’ this could be argued to be ‘NGO speak’, and some critical reflection on this would be useful (i.e. to what extent is ‘grounded experience/discourse’ really *other* to Western discourse in international development contexts/settings?!). Also, the concept of ‘ambiguous agency’ in youth studies speaks to, and challenges, the inherent moralising in mainstream discourses on youth agency, and could be used to connect ‘agency’ more explicitly with the discussion on good vs bad ‘coping’.

We are grateful for this insightful critique, which was extremely helpful; we have expanded on the definition of 'agency' we align with in the conceptual framework and integrated reflections into the discussion.

4. The discussion on safety (p.25-26) doesn’t really fit with the core messages of the paper. Not only does it implicitly centre the individual (and accordingly holds potentials for implicitly blaming individuals) – e.g. can life skills, or employment / access to money really make a person ‘safer’ in these ‘traumatic contexts’? It also seems to suggest that the complex intersecting axes of oppression (and trauma) could be tackled through individual-focussed skills-building interventions and community resourcing/advocacy. Of course, part of the point here is that these traumatic contexts might not be able to be ‘fixed’ through intervention, however one approach which would specifically work to address the different forms of oppression – and would also align with the alternative-to-/more-than-Western/individualistic conceptualisations – would be Paolo Freire’s work.

Thank you for highlighting that our intended message was not translating clearly. We have revised the wording in the paragraph to stress that while the primary need remains tackling the factors of oppression, to obtain this though, parallel interventions are required.

5. A deeper engagement with intersectionality as a lens could also support a richer analysis of the tension (outlined in point 1) – for instance the authors’ positionalities are listed (excepting, oddly, SNC), however not much discussion is provided on the potential limitations of the authors’ range of positioned perspectives. The decolonial concept of ‘locus of enunciation’ (e.g. Decolonial Psychology Editorial Collective (2021). General Psychology Otherwise: A Decolonial Articulation. *ROGP*, 25(4)), for instance, foregrounds the politics of epistemic standpoint, and embodied histories of (dis-/mis-)engagements between knowledge systems. Acknowledging perspectival limitations does not negate the importance of this work; if anything, it strengthens it by fully recognising and engaging with the complexities of knowledge translations.

Thank you for flagging the missing positionalities, this was mistakenly edited out previously and we have re-added. We have also expanded 4.2 ‘Reflections’ section to acknowledge limitations in our interpretive process.

*More minor points*

a) The description of the themes in the abstract is quite convoluted and confusing.

We have edited to improve clarity.

b) I’m not clear on what the difference between sense-making and meaning-making is.

We have expanded on this.

c) In line with point 1 above, the discussion on trauma in the introduction needs to be contextualised (and justified) more. I would suggest bring the discussion on p.25 into the introduction. Also please fully explain all key terms – e.g. continuous traumatic stress.

We have expanded the section on trauma, re-integrating previously removed/moved text, as suggested, along with contextualisation of our approach.

d) Why does specialist knowledge not exist in resource-limited settings (p. 6 line 127)?

Thank you for questioning this; this was not the intended meaning – we have reworded for clarity.

e) More information on the construction of knowledge would strengthen the methods section – e.g. who were the interviewers, how were the character creations supported etc.?

We have added additional information.

f) Shifting the focus of therapeutic interventions from the past to how people navigate the present seems to be one of the core messages of this paper, however it isn’t stated in the abstract, and could be highlighted more throughout the paper.

We have edited the abstract to strengthen.

g) (p.24) Many substance use interventions already do embrace a harm reduction stance.

Yes, we agree, but much of the harm reduction work is focused individually on substance use itself – we are also interested here, in the expansion of harm reduction to consider the wider contexts through which trauma driving substance use exists. We have specified ‘in the context of ongoing trauma’.

h) There are a number of typos e.g. p. 4 line 84, p.5 line 92, p.6 line 113.

We have resolved typos.

**Reviewer 2**

Reviewer #2: A well-written manuscript that sensitively addresses the important topic of trauma among young people in socially disadvantaged settings and provides some really interesting reflections on the limitations of classic interpretations of trauma and PTSD. A few shortcomings in the interpretation of the social context (mainly around description of contemporary South African society, and the glaring absence of a gendered lens) and some minor lack of clarity in presentation of the findings need to be addressed before publication, however.

Thank you so much for taking the time to review our paper and for providing such thoughtful feedback.

*Introduction*
 A more comprehensive definition of ‘trauma’ is needed, with citations. The one currently used (pg 4) is circular and doesn’t tell us much (trauma is the internal response to a traumatic event).

Thank you for flagging this; We have expanded the section and re-added references mistakenly edited out previously.

The absence of mention of gender-based violence in this setting is problematic; “violence” is discussed as if it is almost random but there is a strong gendered element to the forms that violence takes here and who the perpetrators and victims are.

The claim that “apartheid systems of governance and management” ended in 1991 needs qualification. In reality, the dismantling of apartheid legislation took many years following the unbanning of the ANC and the inception of democratic governance in 1994. But to think of “apartheid” only in its narrow sense of racist laws is also problematic because it shaped every aspect of South African society, and even after 1994, this continues to be the case – the most obvious example being in the fact that residential zones set out along racist lines pre-1994 continue to be organised in this way, even if not legislated as such. While the broader argument in the article about the social/structural causes of psychological trauma and the ongoing nature of trauma suggest that the authors understand this point (about apartheid not ending neatly in 1991), it feels disingenuous to make a statement like this and leave it at that.

Thank you for pointing this out – for the sake of length we had removed a longer expansion of both violence and the socio-political context, but we have now re-added it.

*Methods*
 Page 9, line 197 – “the data used in this analysis” is ambiguous. Do you mean the data used in the analysis for the Siyaphambili Youth project? Or the data used in the analysis for this article?

The repetition of certain descriptions of methods used makes for very confusing reading. For example, the in-depth interviews are mentioned in section 2.2 and again in 2.3 (they fit better in 2.3 alone).

Thank you for highlighting this – we have streamlined these sections for greater clarity.

Page 12, lines 233 to 235 – in this description of grouping into themes etc. maybe mention that these are provided as supplementary material?

We have added signposting.

Sub-section on Reflexivity – to be consistent, please provide race and gender details for authors SNC and JM.

Thank you for flagging the missing positionalities, this was mistakenly edited out previously, and we have re-added.

*Findings*
 Here and there, some additional context would help the reader to interpret the participant quotes. For example, page 14, line 287 – “she kept quiet because there is nothing…” - tell us more about who “she” is. In the quote from lines 290 to 294, who is “they” and “she”?

We have added context here.

It’s not entirely clear how the quote on page 15, lines 310-313 illustrate “anticipated social judgment or rejection, and a sense of feeling weighed down by pressures and expectations”. Perhaps provide a second quote to illustrate that point, since the one provided (lines 310-313) only really reflects the lack of physical safety for women.

We have re-structured and added additional quotes to illustrate the findings.

Some of the description on page 17 for the global theme on being mugged comes across as a bit euphemistic – by the phrase “by any means necessary”, are you essentially referring to involvement in crime/theft? Much of the text for this theme is not really convincing. The 2 quotes at the top of page 18 seem to reflect a deliberate choice to be passive and avoid intervening in situations where they witness violence, which suggest an underlying fear of getting involved (as this might be dangerous?) - which doesn’t really resonate with the title of this global theme of “finding ways to obtain resources by any means necessary”, or of being “trapped in the cycle together”.

We have re-written this description to clarify and added an additional quote to illustrate.

It's not clear what is meant by “societal resistance” on page 23, line 480. Resistance to what? Who is resisting?

Thank you for pointing this out - this was mis-phrased, we have corrected it.

*Discussion*
 “These experiences echo Post-Traumatic Growth” – what is Post-Traumatic Growth? In what ways do these experiences echo it? The claim is made without explanation or backing up.

Thank you for flagging this - we have re-added further explanation.

Page 23, line 498 – I take your point that there should be focus on navigating the present, but perhaps “processing the past” should not be jettisoned entirely? I would add “only” just before “processing…”. A similar point could be made of “healing the wounds”, top of page 24.

We have added clarification.

Page 24, line 520 – after “compassionate approach”, add the phrase “in the context of ongoing trauma” – as a way to link your recommendation re harm reduction with your broader argument around trauma without end.

We have updated this wording.

Your points towards the bottom of page 26 about how the young people could adopt “a more empowered stance” beg the question of how they were able to do this amidst what you have described in the article as truly challenging circumstances. What was the “catalyst” that prodded them towards this empowerment? The sentences that follow this raise the possibility that “support systems” and “wider community networks” may have allowed them to “access feelings of acceptance and care”. Is this what is required, then, for people to rise above the oppressive and otherwise disempowering conditions of their social existence in these settings? We don’t really get a sense of how the authors understand “empowerment” and what is needed for it to unfold. We are told what “empowerment” IS – by reference to the text linked with citation #64 – but not how one gets there. It is also not clear whether the intervention (or even just the process of taking part in the study) actually played a role in making this “empowerment” more possible among these young people.

Thank you so much for these thoughtful prompts – we appreciate the opportunity to clarify, and we have expanded on this in the discussion. It was not our intention to imply a need for young people to rise above their circumstances but instead to highlight the significance of everyday acts of agency and resilience that allow individuals to survive and find moments of meaning within challenging environments. That by acknowledging these acts of agency this supports the development of a sense of personal control and self-efficacy – this being a gradual iterative process, which may be facilitated by support systems, access to resources or opportunities to work towards personally meaningful goals such as engaging in community initiatives.

A key gap in the Discussion and in the article overall is the lack of attention paid to gender. There are many references to gender-based violence, and we know that the setting is one where prevalence of GBV is high and culturally, there are some very disempowering ideas about femininity in traditional (and even not so traditional) gender roles. Yet the authors are silent on this, which seems odd. In the Findings, the young people are discussed as if they are a homogenous group, but I would have liked to have seen a disaggregation of experiences at least by gender. Surely the experiences (and coping strategies) for young women are different to those of their male peers?

Thank you for your perceptive observation. We are mindful of this and greatly appreciate the opportunity to clarify. While our initial analyses began to explore gender differences, we made the decision not to focus on this aspect as our primary aim was to understand experiences and identify shared and divergent processes rather than to explore differences specifically. Although the themes are not explicitly gendered due to significant overlap in experiences and strategies, we have added references to gender differences throughout to highlight subtleties in the processes. Unfortunately, our sample size did not allow for a stark differentiation between genders. We agree it would be beneficial for future studies to explore this more specifically, and we have added this point to the reflections section.

Other studies tied to this project have explicitly explored gender differences and local understandings of gender (e.g. Gibbs et al., 2016) and intimate partner violence.

There is mention of “our framework” in the Limitations section, which made me go back and re-read the Discussion to search for this framework. Could the authors consider developing a diagramme to represent their framework?

Thank you for pointing this out - we used this word incorrectly and have reworded for clarity.

*Limitations*
 Page 27, line 578 – “Although discussed within the team” – is this the research team or the team of YRPAs?

We have added clarification and expanded on our reflections in the limitations.

Across the manuscript, there are a number of typos and simple punctuation or grammatical errors that need fixing:
 Pg 4, line 84 – “In casesopen-ended trauma”
 Pg 5, line 92 – “sexist” should be “exist”
 Pg 5, line 93 – “it’s” should not have an apostrophe (only the contraction of “it is” has one, not the possessive “its”)
 Pg 6, line 113 – “synergistc”
 Pg 6, line 125 – remove comma after “context”
 Pg 7, line 131 – replace “be” with “are not”
 Pg 7, line 132 – “evokes” should be “evoke”
 Pg 7, line 151 – remove comma after “distress”
 Pg 8, line 155 – “counter hegemonic” needs a hyphen and remove the comma after “hegemonic”
 Pg 8, line 170 – add a comma after “literature”
 Pg 8, line 171 – replace “their” with “one’s”
 Pg 8, line 174 – add the word “that” after “argues”
 Pg 10, line 198 – replace the word “completed” with “collected”
 Page 10, line 199 – Full-stop after the word “lives”. New sentence beginning “This included…”
 Page 10, line 215 – the word “of” is missing between “experiences” and “overlapping”
 Page 10, line 218 – change “All YPRAs were not in education…” to “None of the YPRAs were in education…”
 Table 1 – Add a footnote to explain that “Matric” is the final year of secondary education.
 Page 12, lines 238-240: Suggested rewording of sentence – “These efforts seek to limit the positioning of researcher perspective as truth over and above the narratives…”
 Page 15, line 303 – place a full-stop after “alive” and start a new sentence with “Maybe”.
 Page 15, line 309 – add the words “and a” between “rejection” and “sense”
 Page 15, line 315 – place a full-stop after “life”, and start the next sentence with “This was illustrated by the fourth global theme…”
 Page 15, line 319 – add the word “believing” – “keeping secrets and believing that it is safer…”
 Page 16, line 334 – add the word “and” after “future” and before “appreciation”
 Page 17, line 344 – amend to “…I was asking [the] ancestors [for the things that] I want…”
 Page 17, line 355 – add a comma and the word “as” between “needs” and “captured”.
 Page 17, lines 358-360 – the sentence beginning “This cycle of shared experience…” doesn’t make sense, grammatically (especially the part at the end – “also alludes to unspoken agreements”.
 Some of the participant quotes need simple punctuation marks (commas and full-stops) to make the meaning clear. For example, page 16, line 323 – “…I can tell that, no, if they were to know, I’d end up…” In the quote below this (lines 326-329), the use of the phrase “jumped off” twice in two different contexts/meanings (?) is a bit confusing.
 Insert quotation marks around “Hello, my girlfriend” and other instances of direct speech in the quote on page 18, lines 380 etc.
 Page 19, line 405 – Remove comma after “from”
 Page 20, line 413 – add “[drugs]” after “high on them”
 Page 20, lines 424-426 – simplify this sentence, which is quite hard to follow
 Page 20, line 430 – the phrase “all stakes rely with the person who is abused…” doesn’t make sense
 Page 21, line 450 – the word “to” is missing between “individuals” and “process”. This sentence is also very long and would be easier to understand if broken up into 2 sentences.
 Page 23, line 486 – Suggested rewording: “…associated with PTSD diagnoses. Instead, they remain in a continuous cycle…”
 Page 23, line 487 – add the word “as” between “successes,” and “young”
 Page 23, line 488 – add the word “being” before “heard”
 Page 23, line 493 – replace “on who” with “to whom”. Add “writers within” before “cultural and critical”
 Page 24, line 506 – add the word “which” between “use” and “while”.
 Page 24, line 507 – remove the comma after “distress”
 Page 24, line 510-511 – put the phrase “can lead to additional harm” right at the end of the sentence – i.e., “…to stop using these strategies, without ensuring that….. can lead to additional harm.”
 Page 24, line 517 – remove the comma after “behaviours”
 Page 25, line 525 – add “and” after “housing” and before “income security”
 Page 25, line 540 – add comma after “settings”
 Page 26, line 553 – end the sentence after “realities”. New sentence beginning with “As such,…”
 Page 26, line 560 – add the phrase “interviewed for this study” after “Young people”
 Page 27, line 576 – add the word “that” after the word “Given”
 Page 27, line 585 – add comma after “low-resource”

Thank you so much for highlighting these – we really appreciate your attention to detail - we have addressed typos and integrated suggestions.
